# Integrative Metabolomics for Assessing the Effect of Insect (*Hermetia illucens*) Protein Extract on Rainbow Trout Metabolism

**DOI:** 10.3390/metabo10030083

**Published:** 2020-02-27

**Authors:** Simon Roques, Catherine Deborde, Laurence Guimas, Yann Marchand, Nadège Richard, Daniel Jacob, Sandrine Skiba-Cassy, Annick Moing, Benoit Fauconneau

**Affiliations:** 1 INRAE, University Pau & Pays Adour, E2S UPPA, UMR NuMeA Nutrition, Metabolism and Aquaculture, F-64310 Saint Pée sur Nivelle, France; simon.roques@inrae.fr (S.R.); sandrine.skiba@inrae.fr (S.S.-C.); benoit.fauconneau@inrae.fr (B.F.); 2 Phileo by Lesaffre, F-59700 Marcq-en-Baroeul, France; n.richard@phileo.lesaffre.com; 3 PMB-Metabolome, INRAE, 2018 Bordeaux Metabolome Facility, MetaboHUB, F-33140 Villenave d’Ornon, France; catherine.deborde@inrae.fr (C.D.); daniel.jacob@inrae.fr (D.J.); 4 INRAE, University Bordeaux, UMR Fruit Biology and Pathology, F-33140 Villenave d’Ornon, France; 5 Copalis Industrie, F-62480 Le Portel, France; l.guimas@copalis.fr; 6 Le Gouessant, F-22402 Lamballe, France; yann.marchand@legouessant.fr

**Keywords:** integrative metabolomics, fish nutrition, insect, proton NMR, profiling, *Oncorhynchus mykiss*, *Hermetia illucens*

## Abstract

Nutrition of high trophic species in aquaculture is faced with the development of sustainable plant-based diets. Insects seem particularly promising for supplementing plant-based diets. However, the complex effect of whole insect meal on fish metabolism is not well understood, and even less is known about insect meal extracts. The purpose of this work was to decipher the metabolic utilization of a plant-based diet supplemented with the gradual addition of an insect protein extract (insect hydrolysate at 0%, 5%, 10% and 15%). ^1^H-NMR profiling was used to assess metabolites in experimental diets and in fish plasma, liver and muscle. A significant dose-dependent increase in growth and feed efficiency with increasing insect extract amounts was observed. The incremental incorporation of the insect extract in diet had a significant and progressive impact on the profile of dietary soluble compounds and trout metabolome. The metabolites modulated by dietary insect extracts in plasma and tissues were involved in protein and energy metabolism. This was associated with the efficient metabolic use of dietary free amino acids toward protein synthesis through the concomitant supply of balanced free amino acids and energy substrates in muscle. The findings provide new insights into how the dietary food metabolome affects fish metabolism.

## 1. Introduction

Fish meal (FM) will no longer be sustainable for aquaculture in the foreseeable future because it is produced from limited stocks of pelagic fish, while the amount of feed used for farmed fish continues to increase [1]. Despite the widespread replacement of FM by plant-based ingredients, full plant-based feeds for high-trophic species still induce negative effects on fish growth and metabolism [2]. Thus, present-day commercial feeds always include FM. However, alternatives to marine resources exist to supplement plant-based feeds devoid of FM, and especially for protein ingredients with the use of single-cell proteins or land-animal proteins [3]. Amongst the sources of land-animal proteins, selected insect species can be incorporated into novel aquaculture diets, the most known being yellow mealworm beetle (*Tenebrio molitor*) and black soldier fly (*Hermetia illucens*). The addition of *H. illucens* larvae in fish feed composition already gives encouraging results on growth performance and is a good nutritional alternative to FM [4,5,6,7]. It has a high protein content, ranging from 40% to 63% of the total biomass depending on the rearing substrate [8], and an adequate amino acid profile quite similar to that of FM, especially regarding sulfur amino acids and lysine, which are limiting in most plant protein ingredients [9]. Protein extracts and hydrolysates are useful dietary ingredients for fish because they supply free compounds that can act as feed attractant, balance the absorption kinetic of amino acids in complete feed, and may contain bioactive peptides [10,11,12,13]. Furthermore, teleosts can specifically benefit of the direct absorption of small peptides in the larval stage and in the later stages, especially through the PepT1 transporter, which is activated during refeeding after a fasting state to ensure compensatory growth as compared to mammals and birds [14,15].

To assess a new ingredient in a feed, key indicators are the palatability of the whole feed, its digestibility and its utilization (growth, feed conversion, nutrient retention, etc.) and even somatic indexes [16]. These classic zootechnical indicators may be used to assess feed efficiency and growth performances but do not give any information about how feed influences these parameters. Omics approaches have led to deeper understanding of the effects of FM substitution in feeds on fish organisms. Nutrigenomics studies [17,18] have revealed several hepatic genes whose transcription is modified by a full plant-based diet in high-trophic species such as rainbow trout (*Oncorhynchus mykiss*) and sea bass (*Dicentrarchus labrax*). Similarly, metabolomic studies have been conducted on high-trophic species to assess the effect of full plant-based diets [19] or of diets based on alternative ingredients such as land proteins or single-cell proteins [20,21]. The combination of classical and omics approaches seems promising to decipher the metabolic mechanisms involved in phenotypic responses.

An integrative approach in nutritional studies should include multiple sources of data at different levels, e.g., feed characterization and fish phenotype, microbiome, transcriptome, proteome and metabolome, to cover the wide range of effects induced by a dietary treatment. By doing so, it could be possible to solve the complexity of effects induced by alternative ingredients used to complement plant-based diets. For example, multiple omic approaches in fish nutritional studies have been combined to assess the relationships between the gut microbiome and the metabolome in rainbow trout as well as in different coastal and estuarine species [22,23], or to link the transcriptome with the metabolome to assess the role of circadian rhythm on the nutritional state of leopard coral grouper (*Plectropomus leopardus*) [24]. However, studies on both feed compounds and fish metabolism by omic approaches are rare. Moreover, we have previously shown that numerous soluble compounds are present in fish feeds and that their characterization could be included in the nutritional integrative approach [25]. For example, Jasour et al. (2018) [26] demonstrated that biogenic amines in fish meal impact the rainbow trout metabolome.

Consequently, we investigated how a plant-based feed supplemented with graded amounts of insect protein extract (*H. illucens*) might affect the growth performance of rainbow trout and its metabolism. Metabolome was characterized in plasma, liver and muscle, using an integrative ^1^H-NMR metabolomic approach. To assess whether metabolic effects and growth performance could be related with the soluble compounds present in the feeds, the main trends of the soluble compounds in the feeds were related to those of the metabolite profiles of fish depending on the amount (0%, 5%, 10% and 15%) of insect extract in the diets. We propose a synthetic vision of the metabolism modifications corresponding to the changes observed in the diets.

## 2. Results

### 2.1. Fish Growth

Initial and final body weights, feed conversion ratio (FCR), daily feed intake, hepatosomatic and viscerosomatic indexes and nitrogen retention are presented in Table 1. Supplementation of the plant-based (PB) diet with the insect extract had a positive and significant effect on fish final body weight (ANOVA, *P*-value *=* 4.10^−4^) with a progressive increase in final body weight depending on the amount of insect extract (INS). The final body weight of fish fed the diet with 5% of insect extract (INS05) was not significantly different from that of the PB diet and the same was observed between fish fed the INS10 and INS15 diets. The final body weight of fish fed the INS10 or INS15 diet was significantly different from that of fish fed the PB and INS05 diets (Tukey’s test, *P <* 0.05). Daily feed intake was significantly modified by diet (ANOVA, *P =* 0.0321): with a lower daily feed intake in fish fed diets supplemented with the insect ingredient (Tukey’s test, *P <* 0.05). Similar effects of the diets were observed on FCR (ANOVA, *P =* 0.0012) i.e., a progressive decrease from PB to INS15 diet with a significant difference between PB-INS05 diets, on one hand, and INS10-INS15, on the other (Tukey’s test, *P <* 0.05). The nitrogen gain was similar between the four diets, but the nitrogen intake decreased when fish were fed insect-supplemented diets (Table S1). Thus, a significant effect (ANOVA, *P =* 0.003) of diet was observed on nitrogen efficiency ratio associated with a significant increase (Tukey’s test, *P <* 0.05) for INS10 and INS15 diets compared to PB diet. Diet had no significant effect on hepatosomatic and viscerosomatic indexes (Kruskal-Wallis, *P =* 0.27 and *P =* 0.18, respectively).

### 2.2. Annotation and Integration of Feed Soluble Compounds and Fish Metabolome NMR Spectra

In ^1^H-NMR spectra of feed extracts, 28 compounds were putatively annotated (Table S2). In spectra of plasma and tissue extracts, a total of 84 metabolites were annotated: 23 in plasma, 36 in liver and 25 in muscle (Table S2). Twenty common metabolites were found in plasma and liver, 16 in plasma and muscle and 24 in liver and muscle. A Venn diagram details the common and specific metabolites within plasma and tissues (Figure S1). The 16 metabolites common to plasma, liver and muscle comprised 13 amino acids or their derivatives, two organic acids and one sugar.

### 2.3. Patterns of Compounds in Feed

**Figure 1:**
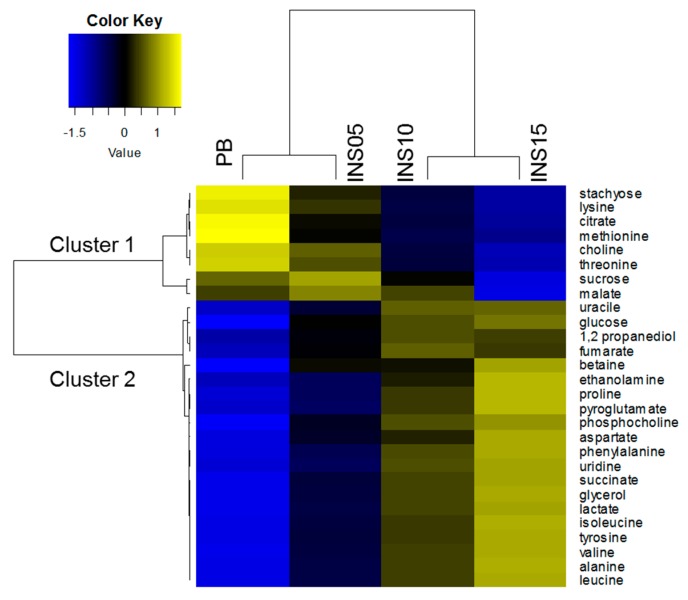
Hierarchical clustering analysis (HCA) heatmap of mean relative intensities of 28 compounds quantified using ^1^H-NMR profiling in feed for control diet (PB) and three diets with increased amounts of insect ingredient (INS05, INS10, INS15).

To assess how compound relative intensities changed in feed with the inclusion of the insect ingredient, a hierarchical clustering analysis (HCA) was performed on compound intensity means per diet. On the double HCA heatmap (Figure 1), the diets were ordinated from PB to INS15 and the feed compounds were grouped into two major clusters. The intensity changes of the compounds of each cluster are plotted in Figure S2. In Cluster 1 comprising eight compounds, the mean compound intensities decreased linearly from PB to INS15 feed. In Cluster 2 comprising 20 compounds, the mean compound intensities increased from PB to INS15 feed but less linearly than in Cluster 1.

### 2.4. Fish Separation Based on Insect Amount in Diet

**Figure 2:**
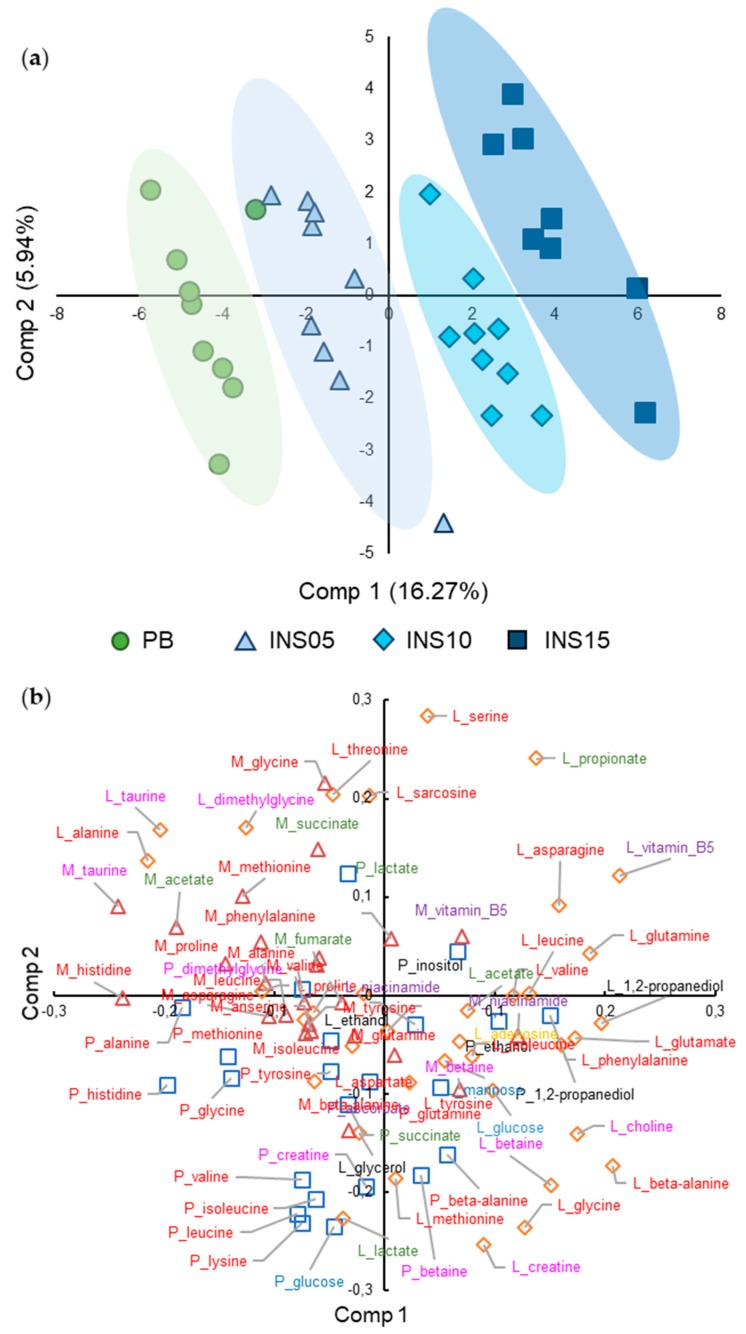
OSC-PLS-DA of 84 metabolites measured in fish plasma, liver or muscle, for fish fed PB, INS05, INS10 or INS15 diet for 84 days. Q^2^ = 0.90, R^2^Y = 90.13%, *P =* 0.036. (**a**) Scores plot of individuals on first two components. Green circles, PB; light-blue triangles, INS05; blue diamond, INS10; dark-blue squares, INS15 diet. (**b**) Loading plots on first two components. Squares, plasma metabolites (P_); triangles, muscle metabolites (M_); diamonds, liver metabolites (L_). All metabolites with a loading value below -0.06 or above 0.06 on one of the first two components are annotated and colored according to their compound family: red, amino acids or dipeptides; green, organic acids; blue, sugars; black, alcohols or cyclitols; pink, amines or N-containing compounds; yellow, nucleobases or nucleosides; purple, vitamins.

Based on the Kruskal–Wallis analysis of the intensities of 84 metabolites from plasma, liver or muscle NMR spectra, 32 metabolites were significantly affected by diet, 7 in plasma, 16 in liver and 9 in muscle (Figure S3). Thus, we analyzed the metabolome for specific changes in metabolites that could be related to linear trends observed for dietary compounds. We used a linear discriminant analysis to identify which metabolite intensities tended to increase or decrease linearly in the fish metabolome. A Partial Least Squares Discriminant Analysis with an Orthogonal Signal Correction (OSC-PLS-DA, Figure 2) was used to discriminate fish based on their metabolomes of plasma and tissues according to the four diets and to identify which metabolite variables were most representative of the fish separation between the four diets. The predictive accuracy and percentage of the variance explained by the diet factor for the OSC-PLS-DA were high: Q^2^ = 0.90 and R^2^Y = 90.1%. The cross-validation *P*-value was significant (*P =* 0.036). As expected, the scores plot (Figure 2a) showed a clear group separation along the first component that concorded with the amount of insect protein extract. Despite the orthogonal signal correction, the diet groups were slightly tilted from a vertical axis. When integrating the scores (Figure 2a) and the loading plots (Figure 2b), overall plasma and muscle metabolites tended to have higher intensities in PB-fed fish, and liver metabolites tended to have higher intensities in INS15-fed fish.

**Figure 3:**
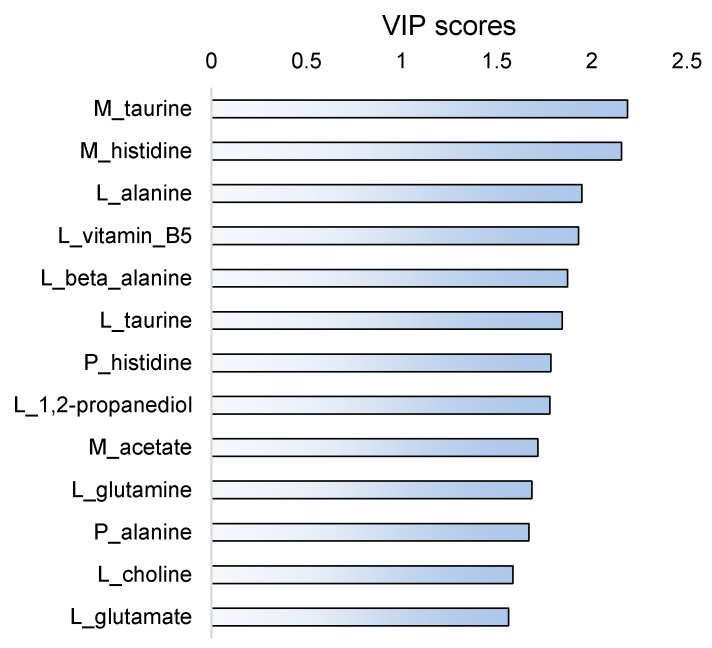
Variable importance in the projection (VIP) scores from the OSC-PLS-DA of 84 metabolites measured in fish plasma (P_), liver (L_) or muscle (M_), for fish fed PB, INS05, INS10 or INS15 diet for 84 days presented Figure 2. The scores values of the 13 variables with a score higher than 1.5 are presented.

A threshold of 1.5 was chosen for the variable importance in the projection (VIP) scores to select the metabolite intensities that contributed most to fish separation according to the diet. Thirteen metabolite intensities had a VIP score higher than 1.5 (Figure 3). Among them, two were from plasma (histidine, alanine), three from muscle (taurine, histidine, acetate), and eight from liver (alanine, vitamin B5, beta-alanine, taurine, 1,2-propanediol, glutamine, choline, glutamate). All these metabolite intensities were significantly affected by the diet (Kruskal-Wallis analysis, *P <* 0.05), with the lowest *P*-value of 0.003 for plasma alanine, except liver choline intensity (*P =* 0.11). Taurine contributed to discriminating diets both in muscle and liver tissues, histidine both in plasma and muscle tissue, and alanine both in plasma and liver tissue. Glutamine and glutamate in liver tissue tended to discriminating diets.

**Figure 4:**
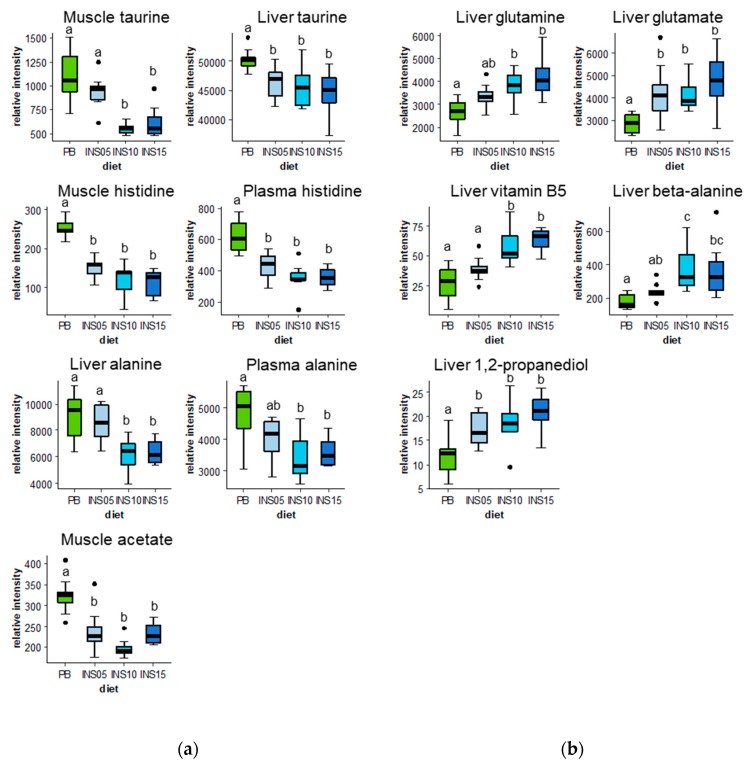
Whisker plots (n = 9 or 8) for 12 fish variables selected using OSC-PLS-DA presented in Figure 2 based on VIP score higher than 1.5 (Figure 3) for diet separation, and significantly affected by diet based on Kruskal-Wallis analysis (*P <* 0.05). (**a**) Fish variables showing a decreasing trend with level of insect ingredient amount in diet. (**b**) Fish variables showing an increasing trend with level of insect ingredient amount in diet. For each plot, medians of diets were compared according to Dunn’s test (*P <* 0.05): diets accompanied by the same letters (a, b or c) above their corresponding whisker plots were not significantly different according to Dunn’s test.

Noticeably, it was possible to plot and classify these metabolites for the four diets (Figure 4) according to two patterns paralleling the patterns highlighted for the feed compounds: gradual decrease (Figure 4a) or increase (Figure 4b) from PB to INS15. Intensities of liver taurine, muscle histidine, muscle acetate and plasma histidine were significantly reduced in INS-fed groups compared to the PB-fed group. Muscle taurine and liver alanine intensities were significantly lower in INS10 or INS15 groups than in PB and INS05 groups. Liver glutamate and liver 1,2-propanediol intensities were significantly higher in INS groups than in the PB group. Liver vitamin B5 and liver beta-alanine intensities were significantly higher in INS10 and INS15 groups than in PB and INS05 ones.

Diet had a significant effect on the mean of the sum of six of the free essential amino acids in fish muscle (ANOVA, *P =* 5.4.10^−5^) measured using the intensities of the identified corresponding amino acids on the zgpr NMR spectra (Figure S4). The intensity sum of all these free essential amino acids identified and quantified were significantly lower in INS15 and INS10 fed groups compared to PB, while that of INS05 did not differ from PB (Tukey’s test, *P <* 0.05).

## 3. Discussion

This study was conducted to assess the effect of supplementing an insect protein extract to a plant-based diet on the metabolome of rainbow trout. The hypothesis was that the numerous soluble compounds of the insect extract ingredient could act either as attractant to stimulate feed intake or metabolically to improve fish growth performances fed plant-based diets.

Based on the growth performances (Table 1), the insect protein-extract ingredient significantly increased final body weight and improved feed efficiency and nitrogen retention efficiency. However, the daily feed intake did not increase but significantly decreased from PB to INS15, demonstrating that the soluble compounds of the insect ingredient in the feed had no effect on the attractiveness of the diet. Other factors such as energy content or nutrient availability in feed usually drive feed intake [27]. The experimental diets were iso-energetic while free amino acid contents were higher in INS diets. Thus, it is suggested that the supply of nutrients as free form from INS diets covered the nutrient requirements faster than with PB diets. This contributed to the increased protein retention efficiency and probably explained the decreased feed intake also. Therefore, this offered a unique opportunity to analyze the changes induced by insect diets on metabolite patterns by assessing the potential metabolic effect of soluble compounds found in the diet.

### 3.1. Protein Synthesis-Oriented Metabolism Induced by Dietary Compounds

**Figure 5:**
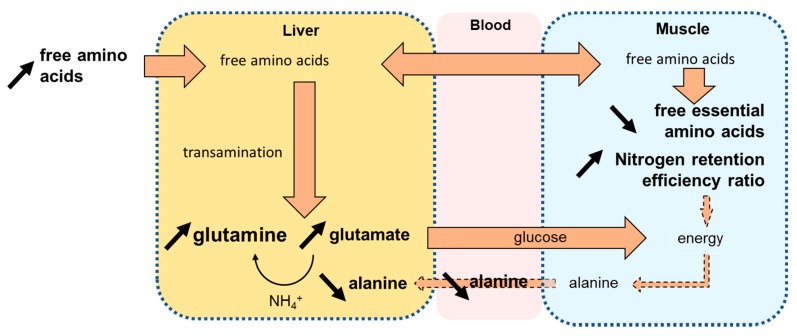
Simplified diagram of the relationships between liver, blood and muscle metabolites involved in Cahill cycle and protein synthesis, integrating hypotheses based on the present experiment. The increase of free amino acids from INS diet would rise the glutamate and glutamine contents in liver to supply energy to muscle. In muscle, the free amino acids are rather used for protein synthesis than for energetic purpose. The alanine content from Cahill cycle would consequently decrease.

An interesting finding is the clear amino acid and protein metabolic status in trout, which could be deduced from the metabolite pattern of glutamate, glutamine in liver and alanine in liver and plasma combined with the decrease in free essential amino acids content in muscle. In carnivorous fish, amino acids not only constitute building blocks for protein synthesis in tissues but are also used as preferential energetic substrates together with fatty acids [28]. As energetic substrate, amino acids are catabolized by transamination in liver, except for the branched-chain amino acids which are mostly catabolized in muscle. Transamination transfers the amine function from an amino acid to an α-ketonic acid that generates glutamate and a novel α-ketonic acid. In the present study, the glutamate intensities in liver increased consistently with the ratio of insect extract in feed, which suggests a predominant amino acid catabolic activity for higher energy supply. Glutamate is then converted back to α-ketoglutarate by glutamate dehydrogenase, a step that produces free ammonia. However, even though teleosts can process and excrete high levels of ammonia directly, too high a concentration results in harmful effects [29]. Thus, ammonium can be fixed on glutamate to form glutamine through glutamine synthetase and alleviate such effects [30]. The concomitant rise in glutamine intensities observed in our study in the liver of fish fed the diet containing a high level of insect ingredient is consistent with this effect. In this case, increased glutamate and glutamine intensities in liver indicate an increase in amino acid catabolism for energy supply (Figure 5). This process might be fueled by the continuous supply of both free amino acids from the insect extract and the delayed release of amino acids from protein digestion. As we did not identify any changes in aspartate or asparagine intensity, the glutamate may be a privileged pathway to fuel the TCA cycle.

While there was a relative hepatic increase in glutamate and glutamine intensities, a decrease in alanine intensity was observed in liver and plasma. Alanine may be considered as one of the end-products of amino acid catabolism in muscle. The amino acids in excess in muscle during fasting or active swimming activity are further transaminated on pyruvate to produce alanine. Alanine is then transported through the blood to the liver, thus contributing to the Cahill cycle between muscle and liver (Figure 5). In fish fed insect extracts from 0% to 15% (PB to INS15), a decrease in alanine intensity was observed both in plasma and liver, which suggests that amino acid catabolism is reduced in the muscle of fish fed insect extract-supplemented diets.

The muscle metabolism seems to be preferentially oriented toward protein synthesis in fish fed diets supplemented with insect extract, as suggested by the reduced accumulation of free essential amino acids in muscle (Figure S4) and the increased whole-body nitrogen retention efficiency (Table 1). This protein metabolism state might explain the reduced levels of alanine observed in liver and plasma, which serve as a marker of reduced amino acid catabolism. This metabolism oriented towards protein synthesis in muscle might be favored by the significant supply of free amino acids and peptides from insect protein extracts, which are rapidly absorbed and transported to muscle compared to plant-based diets, in which the absorption of amino acids is generally delayed [31]. Furthermore, the increased glutamate and glutamine levels in liver indicate the increased use of amino acids to supply the energy required for protein synthesis in muscle.

### 3.2. Metabolites as Markers of Alteration of Metabolism Induced by PB Diet

The decrease in taurine intensity in muscle and liver of fish fed the INS diet could partly be due to the profile of the soluble compounds of insect feed. Taurine is a precursor of biliary acids for lipid emulsification which facilitate their digestion. It is also involved in detoxification processes in liver and in osmoregulation, especially in muscle and heart [32]. Although rainbow trout can synthesize taurine *de novo*, it is considered as a semi-essential nutrient in rainbow trout, because a dietary taurine deficiency may degrade growth performance of teleosts [32]. It has been shown that trout hepatic and muscle taurine are a good marker of the level of taurine in the diet [33]. However, plant ingredients are not known to contain taurine, unlike animal feed ingredients [34]. Although insects may synthesize taurine, black soldier fly larvae meal contains very low amounts of taurine compared to fish meal [35]. Furthermore, taurine was not supplemented in our diets (Table 2), and no differences in taurine were observed in the feed profiling of soluble compounds. Thus, taurine in fish tissue must come exclusively from *de novo* synthesis. In rainbow trout, the main pathway for taurine synthesis is not yet clearly established, but most evidence indicates that synthesis goes through cysteine to form cysteine sulfinic acid, then hypotaurine, and finally taurine [32]. Cysteine is a non-essential sulfur-containing amino acid supplied either directly by ingredients in the diet or synthesized from methionine. However, diet formulation was done to ensure that total methionine and also cysteine contents were not different in the experimental diets. Thus, the relative predominance of taurine in the tissues of fish fed PB feeds could be due to the stimulation of taurine synthesis to ensure its specific functional properties [32]. The anti-nutrient factors of plant ingredients such as sucrose and stachyose oligosaccharides detected in PB diets are known to be osmotically active in the fish gut [36] where they alter both gut structure and function. Consequently, ion absorption might be altered, thus triggering the accumulation of taurine in liver and muscle. However, further information on the alteration of gut ion absorption in fish fed plant-based diet is required to strengthen this hypothesis.

Histidine also decreased in plasma and muscle in response to insect protein extract supplementation. Gomez-Requeni et al. (2004) [37] also found that free histidine in muscle gradually decreased with the reduction of plant ingredients in feed for a carnivorous fish. Thus, this seems to confirm that histidine changes in fish metabolome are not related to a specific alternative ingredient but rather to the decrease in plant protein ingredients. As histidine is a compound that shares the same pH buffering and metal-chelation properties as its related peptides anserine and carnosine, we suggest that an imbalanced pH homeostasis induced by the PB diet was progressively changed by insect protein extract supplementation. The increasing levels of beta-alanine in liver as the amount of insect ingredient increased suggests that the status of carnosine, another related peptide involved in buffering capacity, is also modified.

Amongst the metabolites that demonstrate significant changes with insect protein extract complementation, some compounds such as vitamin B5 and 1,2-propanediol are interesting markers of insect ingredient composition. The annotation of the NMR signal corresponding to vitamin B5 requires further investigation, however the progressive improvement in vitamin status may contribute to the improvement of nutritional status in fish fed the INS diet. Although 1,2-propanediol could be considered as a potential energy substrate in fish, it is difficult to establish from its accumulation in liver of fish fed insect extract whether it plays any functional or metabolic role in fish.

## 4. Materials and Methods

### 4.1. Growth Trial and Sampling

Three hundred sixty female juvenile rainbow trout (49.10 ± 1.05 g average body weight) from INRAE experimental fish farm facilities (INRAE, UMR Nutrition, Metabolism and Aquaculture, F-64490 Lees-Athas, F-40360 Donzacq, France) were reared in 100 L tanks of a flow-through system supplied with water at constant temperature 17 ± 1 °C and constant oxygen level. Fish were split randomly into 12 groups of 30 fish. The fish were fed for 84 days either a full plant-based feed (PB) or plant-based feeds supplemented with 5%, 10% and 15% of insect ingredient (INS05, INS10 and INS15, respectively), i.e., three replicate tanks per diet. Fish were hand-fed twice a day until visual satiation. For each group, the biomass and the amount of distributed feed were measured every three weeks and the mean final body weight was calculated by dividing the total biomass weight by the number of fish alive in each tank. Nine fish per diet (3 per tank) were sampled 48 h after the last meal. Fish were sedated by immersion in a tank containing 10 mg/L benzocaine solution, then anaesthetized by immersion in a benzocaine solution of 30 mg/L. Then blood samples were collected with heparinized syringes and poured into 2 mL Eppendorf tubes. Each tube was centrifuged (3000× *g*, 5 min) and 800 µL of plasma were collected in a 2 mL Eppendorf tube, immediately stored at −20 °C until the end of sampling, then further stored at −80 °C until sample preparation for NMR analysis. Just after the blood sampling, fish were euthanized by section of the spinal cord. The liver was collected and the gallbladder was delicately removed. The liver was rinsed in a saline solution (NaCl 9 g/L), dried on a wrapped paper then immediately weighed and frozen in liquid nitrogen. The whole digestive tract was removed manually and weighed. A portion of 2–3 g from the dorsal epaxial white muscle was sampled just below the dorsal fin after removal of the skin and the red muscle and frozen in liquid nitrogen. Liver and muscle samples were stored at −80 °C until sample preparation for NMR analysis. The ratio between liver mass to fish mass or gut mass to fish mass was calculated.

Both growth trial and sampling were in strict accordance with (i) EU legal frameworks concerning the protection of animals used for scientific research (Directive 2010/63/EU), (ii) the National Guidelines for Animal Care of the French Ministry of Research (decree n°2013-118, February 1st, 2013), and (iii) the local ethical committee “Comité d’Ethique en Expérimentation Animale Aquitaine Poissons Oiseaux” (C2EA-73). The trial did not need specific ethical approval because it involved standard rearing practices and diets formulated to cover the nutritional requirements of rainbow trout. All the staff of the experimental facility received training and personal authorization (N°B64 10 005).

### 4.2. Diet and Fish Whole Body Composition Analysis.

The feed preparation was described in Roques et al. (2018) [25]. All diets were isoproteic, isolipidic and isoenergetic. The control diet was exclusively based on plant ingredients (PB) so it was devoid of FM and fish oil, and was supplemented with graded amounts (0%, 5%, 10% and 15%) of an insect protein hydrolysate ingredient from defatted larvae of black soldier fly (*H. illucens*) (PB, INS05, INS10, INS15, respectively) (Table 2) described previously [25].

Proximate analysis of the experimental diets and measurement of whole-body composition were performed as follows. Dry matter was analyzed by drying the samples to constant weight at 105 °C for 24 h. Crude protein was determined using the Kjeldahl method after acid digestion and estimated by multiplying total nitrogen by 6.25. Crude lipid content was quantified by petroleum diethyl ether extraction using the Soxhlet method. Gross energy content was determined in an adiabatic bomb calorimeter (IKA). Ash was examined by combustion in a muffle furnace at 550 °C for 16 h.

Daily feed intake as percentage of body weight was calculated as follows:

$$\frac{F_{d}\times 100}{\left(\frac{BW_{i}+BW_{f}}{2}\right)\times n_{j}}$$

where *F_d_* is the feed consumed during the growth period, *BW_i_* and *BW_f_*, the initial and final average body weight, respectively and *n_j_* the number of days of trial. The feed conversion ratio, FCR, represents the efficiency of the transformation of feed to biomass and was calculated as follows:

$$\frac{F_{d}}{BW_{f}+BW_{d}-BW_{i}}$$

where *BW_d_* is the total biomass of the dead fish.

Nitrogen intake was calculated as follows: feed intake x nitrogen content in diet. Nitrogen gain corresponds to the difference of nitrogen in fish (fish body weight x nitrogen content in fish) between the end of the experiment and the beginning divided by the duration of experiment in days. The nitrogen efficiency ratio is the ratio between nitrogen gain in fish and nitrogen intake from feed during the experiment.

### 4.3. Sample preparation

For feed samples, freeze-dried feed powders were extracted according to Roques et al. (2018) [25] in five replicates per feed. Briefly, a series of hot ethanol/water extractions was performed, followed by steps of lyophilization and solubilization with deuterated phosphate buffer solution and pH adjustment to apparent pH 6.00 ± 0.02 by means of a titration robot (BTpH, Bruker, Kalrsruhe, Germany). Lyophilized pH-adjusted feed extracts were dissolved again in deuterated water containing deuterated ethylene tetra-acetic acid disodium salt (EDTA-*d_12_*) to chelate paramagnetic cations and (trimethylsilyl)propionic-2,2,3,3-*d_4_* acid sodium salt (TSP) was used for chemical shift calibration just before NMR spectra acquisition.

All fish were still juvenile female, except one male that was discarded from sample preparation. Plasma samples were individually thawed on an ice bed for a minimum of 2 h and no more than 2 h and 20 min, then 300 µL were transferred to an Eppendorf tube and diluted with 300 µL D_2_O (99.8%, Eurisotop, Gif sur Yvette, France). A simple dilution with a lock substance is suitable in liquid ^1^H-NMR to efficiently assess metabolites as a specific acquisition sequence (see Section 4.5) can be used to reduce the signals from macromolecules [22]. The tube was vortexed for 5 s maximum and 550 µL were transferred to a 5-mm NMR tube (Wilmad, Vineland, USA). No TSP was added to the plasma sample because proteins of the native plasma could bind to TSP and shift the -CH_3_ resonance from 0 ppm. NMR acquisition was performed immediately after each sample preparation.

For liver and muscle samples, polar metabolites were extracted using phase separation based on methanol/chloroform/water recommended by Lin et al. (2007) [38]. In our case however for safety reasons the chloroform was substituted by dichloromethane which share similar extraction efficiency. The extraction solvent was a mix of dichloromethane (Honeywell Riedel-de Haën, Puriss ACS, Charlotte, North Caroline, USA) and methanol (Carlo Erba, RPE ACS, Chaussée du Vexin, France) (2:1 v:v) with 0.01% of butylhydroxytoluene as antioxidant (Sigma-Aldrich, Saint-Louis, Missouri, USA). A sample of 1.4 ± 0.2 g frozen tissue was weighed and immediately transferred to a 50-mL Falcon tube on an ice bed. Fifteen mL of the extraction solvent were poured into the 50-mL Falcon tube containing the frozen tissue. The tissue was ground with an Ultra-Turrax T25 disperser (IKA, Staufen, Germany) for 1 min at 9500 rpm, then the homogenate was filtered with two overlaid Whatman filter papers on the Büchner through vacuum filtration. The residual powder was resuspended in 15 mL of extraction solvent and the process repeated once. The combined filtered solutions were mixed with 7.5 mL of aqueous NaCl (0.73% w/v) to enhance the phase separation and were allowed to decant for 10 min minimum. They were then centrifuged at 2200× *g* for 10 min (AVANTI J-20 XP centrifuge, Beckman Coulter, Brea, California, USA). Fifteen mL of the polar phase were then collected and stored at −20 °C until preparation for NMR acquisition.

To prevent a biased estimation of metabolite content, the volume of the polar phase collected was carefully adjusted to the weight of frozen tissue extracted in order to obtain the same final dried matter in each polar extract. The polar phase was transferred to a glass tube (Pyrex disposable culture tube, Mexico), then dried under a moderate nitrogen flow (ALPHAGAZ 1 Azote SMARTOP, Air Liquide, Paris, France) until visual dryness. Dry extracts were solubilized in 140 µL of a deuterated 200 mM potassium phosphate buffer solution containing 2 mM EDTA-*d_12_* (Sigma-Aldrich, Saint-Louis, Missouri, USA), vortexed for a few seconds, then transferred to a 2-mL plastic Eppendorf tube with a Pasteur pipette. Each glass tube was rinsed with 230 µL, then 330 µL D_2_O. The buffered extract was vortexed for a few seconds, then pH was adjusted to apparent pH 6.00 with NaOD or DCl using a titration robot (BTpH, Bruker, Kalrsuhe, Germany). Finally, 550 µL of pH-adjusted extract were transferred to a 5 mm NMR tube (Wilmad, Vineland, USA) with 5 µL of TSP-*d_4_* (98% D) from Sigma-Aldrich (Paris, France).

### 4.4. Total Amino Acids in Feeds

Total amino acid profiles of feeds were determined by targeted ion-exchange HPLC-UV analyses of feed hydrolysates performed by Upscience (Vannes, France) according to the NF standards EN ISO 16634-1 and are presented as % of feed.

### 4.5. NMR Acquisition

NMR acquisition was performed in 5-mm NMR tubes using a Bruker Avance III 500 MHz spectrometer (Bruker, Wissembourg, France) equipped with a sample holder and an ATMA broadband inverse probe. 1D NMR acquisition parameters for feed extracts, have been detailed previously [25].

For plasma, 1D Carr-Purcell-Meiboom-Gill (CPMG) ^1^H-NMR spectra with presaturation (cpmgpr1d) were acquired with 8 dummy scans before 64 scans of 32K data points, a 90° pulse angle, a 6000 Hz spectral width with 2.73 s acquisition time and 2 s recycle delay. A 200 µs echo time and 150 loops were used as parameters for the CPMG experiment. A 5.10^−5^ W irradiation power was used for presaturation.

For liver and muscle, 1D ^1^H-NMR spectra of tissue extracts with presaturation (zgpr) were acquired with 32 scans of 64K data points, a 90° pulse angle, a 6000 Hz spectral width with 5.46 s acquisition time and 10 and 25 s recycle delay for liver and muscle, respectively. A 7.10^−5^ and 8.10^−5^ W irradiation power was used for presaturation for liver and muscle, respectively. Two-dimensional experiments, J-resolved (Jres), ^1^H-^1^H homonuclear correlated spectroscopy (COSY) and ^1^H-^13^C heteronuclear single-quantum correlation (HSQC), were performed on selected samples to help for the annotation of ambiguous signals.

### 4.6. NMR Spectra Processing

Spectra were processed in groups of sample type with the NMRProcFlow tool [39]. Each spectrum was first zero-filled to four times their data points or twice for muscle. Free induction decay (FID) was then multiplied by an exponential window function with a line broadening of 0.3 Hz and automatically zero-order phased. For feed and tissue extracts, the spectra were calibrated on -CH_3_ of TSP-*d_4_* (δ 0 ppm), and for plasma on -αCH of glucose (δ 5.22 ppm). A representative 1D ^1^H-NMR spectrum of feed extracts, plasma, and liver or muscle extracts was annotated based on Roques et al. (2018) [25], Kullgren et al. (2010) [40], Shen et al. (2018) [41] and chemical shift, signal multiplicity and intensity ratio were compared to an in-house database, a public database (HMDB, http://www.hmdb.ca) and the ChenomX NMR Suite library 8.3 (ChenomX Inc., Edmonton, Canada). Metabolite annotation followed the recommended guidelines of Sumner et al. (2007) [42]. All full spectra of feed, plasma or tissues were manually corrected for baseline and uncontrolled peak shifts. A variable size spectral region, or bin, was defined on one well-resolved resonance for the relative quantification of each metabolite, on the full pattern whenever possible or on resolved peaks of the pattern, as detailed in Table S1. These spectral regions were then normalized to constant sum for each dataset. Each integrated spectral region was named after the compound/metabolite it corresponded to, preceded by the letter of the sample type (F: feed, P: plasma, L: liver, M: muscle).

### 4.7. Statistical Analysis

For the physiological parameters of fish, a one-way ANOVA was carried out with R statistical software (v.3.6.1, R Development Core team, 2008) to assess the effect of diet on fish final body weight *BW_i_*, feed conversion, feed intake and nitrogen efficiency. When the *P*-value was lower than 0.05, Tukey’s test was carried out for diet mean comparisons. Similarly, the Kruskal–Wallis test was used to assess the effects of hepatosomatic and viscerosomatic index on the fish sampled for metabolomics analysis (n = 9 per diet).

For feed characterization, before performing the hierarchical clustering analysis (HCA) of feed soluble compound ^1^H-NMR data, the normalized values of bins were mean-centered and scaled to unit variance. The normalized and scaled values of all bins were used to calculate the relative mean intensity of compound per diet (n = 5 for experimental diet except for PB feed n = 4, where one sample was removed due to contamination detected during extraction). The HCA was then computed using R statistical software with Pearson correlation distance and average linkage.

For fish metabolomic data, prior to the Orthogonal Signal Correction Partial Least Squares Discriminant Analysis (OSC-PLS-DA), the data from plasma, liver and muscle were combined into one dataset. One sample of plasma was missing so the Non-linear Iterative Partial Least Squares (NIPALS) algorithm was used to impute the corresponding values. The bins were then mean-centered and scaled to unit variance. The imputation and OSC-PLS-DA were performed with BioStatFlow web software (v.2.9 http://www.biostatflow.org) on the combined dataset. A 200-fold cross validation (leave one out) was used to assess the significance (*P*-value) of the prediction and separation parameters (Q^2^ and R^2^Y). A threshold of 1.5 was chosen for the selection of variable importance in the projection (VIP). The diet effect was then tested on these variables with the Kruskal–Wallis test (*P <* 0.05) and Dunn’s test (*P <* 0.05) for mean comparisons performed with R statistical software.

Based on the NMR annotation, we estimated the sum of free essential amino acids by calculating the sum of relative intensities of six of the free essential amino acids identified in the muscle for each fish to assess if fish tended to accumulate the free essential amino acids. The mean of the sum of free essential amino acids was calculated for each diet group (n = 9, except n = 8 for group INS15 where a male was discarded). A one-way ANOVA was used to determine whether diet had a significant effect on the mean of the sum of free essential amino acids in muscle. When the *P*-value was lower than 0.05, Tukey’s test was performed for diet mean comparisons.

## 5. Conclusions

Integrative ^1^H-NMR-metabolomics on both diet and fish have helped to identify the main underlying mechanisms contributing to fish performance. It thus appears that the insect (*H. illucens*) protein-hydrolysate tested in this work is a valuable alternative protein source to substitute plant-ingredients in fish diet for the improvement of fish performance in fish. Indeed, this ingredient increased the protein efficiency ratio without any increase in feed intake. The supply of higher level of free dietary amino acids and peptides by insect protein extract provides substrates for both protein synthesis-oriented metabolism and the associated energy requirement. This was strengthened by the glutamate and glutamine increase in liver, consistently with the reduced accumulation of essential free amino acids in muscle and reduced hepatic and circulating alanine. Likewise, dietary modulations could be also viewed through significant improvement of important metabolite status, as with taurine and histidine. Such an integrative approach improves the understanding of the metabolic pathways and processes involved in fish metabolism. It is a promising tool to improve new fish feeds formulated with alternative ingredients.

## Figures and Tables

**Table 1:** Growth and feed intake data for trout fed plant-based (PB) diet or diets with increasing amounts of insect ingredient (INS05, INS10 and INS15) for 84 days. Mean ± standard deviation. n = 3 tanks with 30 fish in each tank, or n = 9 fish for each diet.

Zootechnical Parameters	PB	INS05	INS10	INS15
Initial body weight (g) †	48.67 ± 1.35	48.90 ± 1.01	48.90 ± 1.01	48.90 ± 1.01
Final body weight (g) †	247.33 ± 13.99 ^a *^	258.67 ± 11.57 ^a^	293.56 ± 5.35 ^b^	303.33 ± 8.33 ^b^
Daily feed intake (% of body weight per day) †	1.57 ± 0.03 ^a^	1.50 ± 0.03 ^ab^	1.51 ± 0.03 ^ab^	1.48 ± 0.03 ^b^
Feed conversion ratio†	0.98 ± 0.03 ^a^	0.92 ± 0.03 ^ab^	0.89 ± 0.02 ^bc^	0.86 ± 0.02 ^c^
Nitrogen efficiency ratio (N retention % of N intake) †	40.32 ± 1.55 ^a^	44.68 ± 1.58 ^ab^	46.47 ± 2.48 ^b^	48.66 ± 1.06 ^b^
Hepatosomatic index ‡	0.77 ± 0.12	0.87 ± 0.11	0.84 ± 0.19	0.84 ± 0.9
Viscerosomatic index ‡	8.96 ± 1.37	9.77 ± 1.34	9.09 ± 0.58	8.74 ± 0.85

† data generated from pooled fish (n = 3 tanks of 30 fish each); ‡ data generated at end of trial from fish used for the metabolomics approach (n = 9 individual fish); * For final body weight, daily feed intake, feed conversion ratio (FCR) and nitrogen efficiency ratio, means accompanied by the same letter are not significantly different (Tukey’s test, *P >* 0.05).

**Table 2:** Diet formulation (g.100 g^−1^) of control diet (PB) and experimental diets (INS05, INS10, INS15). Ingredients and gross energy.

Ingredients	PB	INS05	INS10	INS15
DHA*-rich algae meal	6.84	6.84	6.84	6.84
Insect protein hydrolysate		5.00	10.00	15.00
Vegetable oils ^(a)^	18.10	16.95	16.00	14.95
Plant proteins ^(b)^	70.40	66.74	62.78	58.79
Rapeseed lecithin	1.00	1.00	1.00	1.00
Monocalcium phosphate	1.20	1.10	1.00	1.00
Phytase	0.02	0.02	0.02	0.02
Lysine 78%	0.50	0.50	0.50	0.50
DL-methionine 98%	0.65	0.56	0.57	0.61
Threonine 98%	0.20	0.20	0.20	0.20
Vitamin premix	0.30	0.30	0.30	0.30
Vitamin C monophosphate 35	0.04	0.04	0.04	0.04
Mineral premix	0.30	0.30	0.30	0.30
Liquid choline	0.15	0.15	0.15	0.15
Antioxidant	0.15	0.15	0.15	0.15
Antifungal	0.15	0.15	0.15	0.15
**Energy** (kJ.g^−1^ dry matter)	24.03	24.50	24.59	24.71

*DHA, docosahexaenoic acid; (a) Vegetal oils including rapeseed oil and linseed oil; (b) Plant proteins including wheat gluten, hydrolyzed wheat gluten, pea protein, faba bean protein concentrate, soy concentrate, soybean meal, rapeseed meal, peeled faba bean and wheat.

## Supplementary Materials

The following are available online at https://www.mdpi.com/2218-1989/10/3/83/s1, Figure S1: Venn diagram of metabolites identified in plasma and tissues; Figure S2: Mean of all compounds of each major cluster of Figure 1 HCA for four diets.; Figure S3: Metabolites in plasma, liver or muscle for which diet had statistically significant effect; Figure S4: Mean of sum of free amino acids calculated from ^1^H-NMR integration in muscle; Figure S5: Representative 500 MHz ^1^H-NMR spectra of plasma of trout fed INS15 diets; Figure S6: Representative 500 MHz ^1^H-NMR spectra of liver polar extract of trout fed INS15 diets; Figure S7: Representative 500 MHz ^1^H-NMR spectra of muscle polar extract of trout fed INS15 diets; Table S1: Amount of feed consumed during fish growth period, nitrogen intake and nitrogen gain; Table S2: Annotation table of feed compounds and metabolites in plasma, liver and muscle; Table S3: MSI levels for annotated compounds and metabolites; Table S4: Total amino acid (free amino acids and protein amino acids) concentrations in the four feeds.
