# Supplementary material for: Integrative Metabolomics for Assessing the Effect of Insect (Hermetia illucens) Protein Extract on Rainbow Trout Metabolism
Source: Metabolites. 2020 Feb 27;10(3):83. doi: 10.3390/metabo10030083 (PMC7143670; doi:10.3390/metabo10030083)
Supplement: Supplementary file 1 [file metabolites-10-00083-s001.pdf]

Integrative metabolomics for assessing the effect of insect (*Hermetia illucens*) protein extract on rainbow trout metabolism

Simon Roques[1,2,3], Catherine Deborde[3,4], Laurence Guimas[5], Yann Marchand[6], Nadège Richard[2], Daniel Jacob[3,4], Sandrine Skiba-Cassy[1], Annick Moing[3,4*], Benoît Fauconneau[1]

[1] INRAE, Univ Pau & Pays Adour, E2S UPPA, UMR 1419, Nutrition Métabolisme, Aquaculture, Saint Pée sur Nivelle, 64310, France ; simon.roques@inrae.fr, sandrine.skiba@inrae.fr, benoit.fauconneau@inrae.fr
[2] Phileo by Lesaffre, 59700 Marcq-en-Baroeul, France; n.richard@phileo.lesaffre.com
[3] Bordeaux Metabolome Facility, MetaboHUB, Centre INRAE de Nouvelle Aquitaine Bordeaux, 33140 Villenave d'Ornon, France
[4] INRAE, Univ. Bordeaux, UMR 1332 Fruit Biology and Pathology, Centre INRAE de Nouvelle Aquitaine Bordeaux, 33140 Villenave d'Ornon, France ; annick.moing@inrae.fr, catherine.deborde@inrae.fr, daniel.jacob@inrae.fr
[5] Copalis Industrie, 62480 Le Portel, France, l.guimas@copalis.fr
[6] Le Gouessant, 22402 Lamballe, France ; yann.marchand@legouessant.fr

* Correspondence: annick.moing@inrae.fr; Tel.: +33-5-57-12-25-28

Received: date; Accepted: date; Published: date

Supplementary Materials

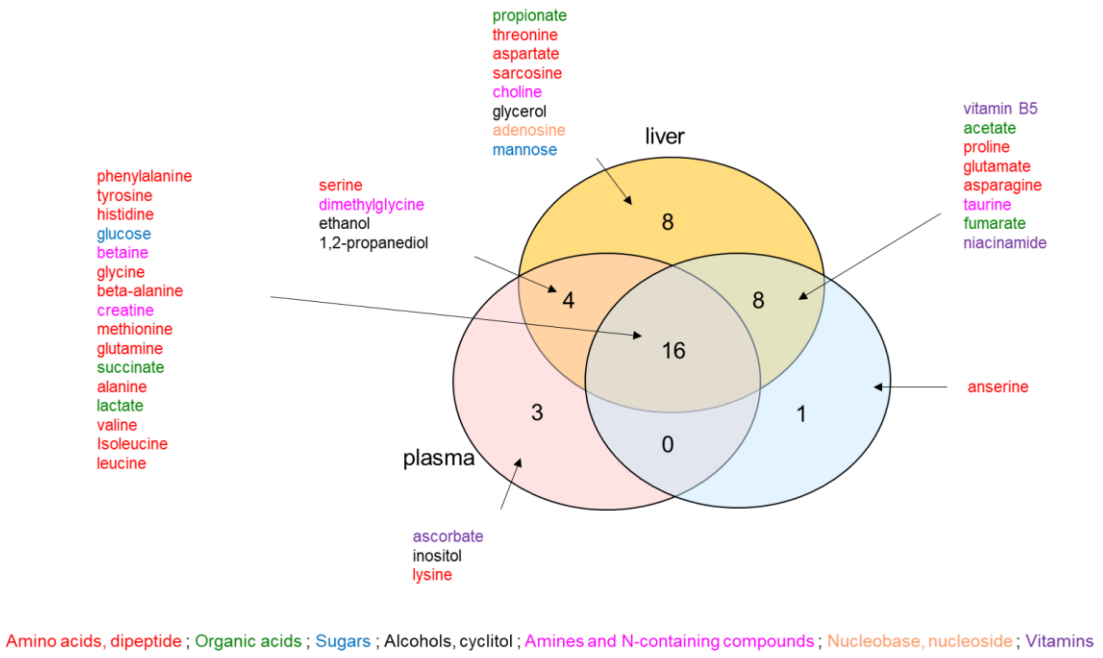

Figure S1. Venn diagram of metabolites identified in trout plasma and tissue polar extracts using NMR.

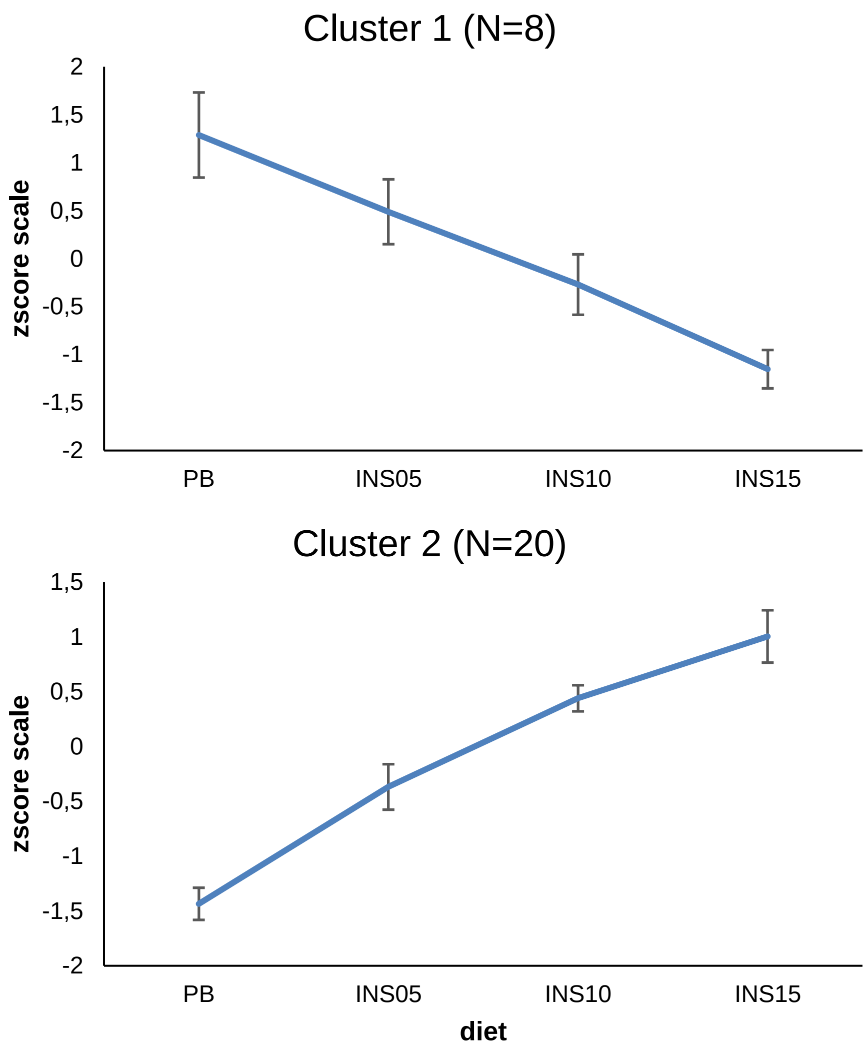

Figure S2. Mean of all compounds of each major cluster of Figure 1 HCA for four diets. Vertical bars represent standard deviation. N indicates number of compounds in each cluster.

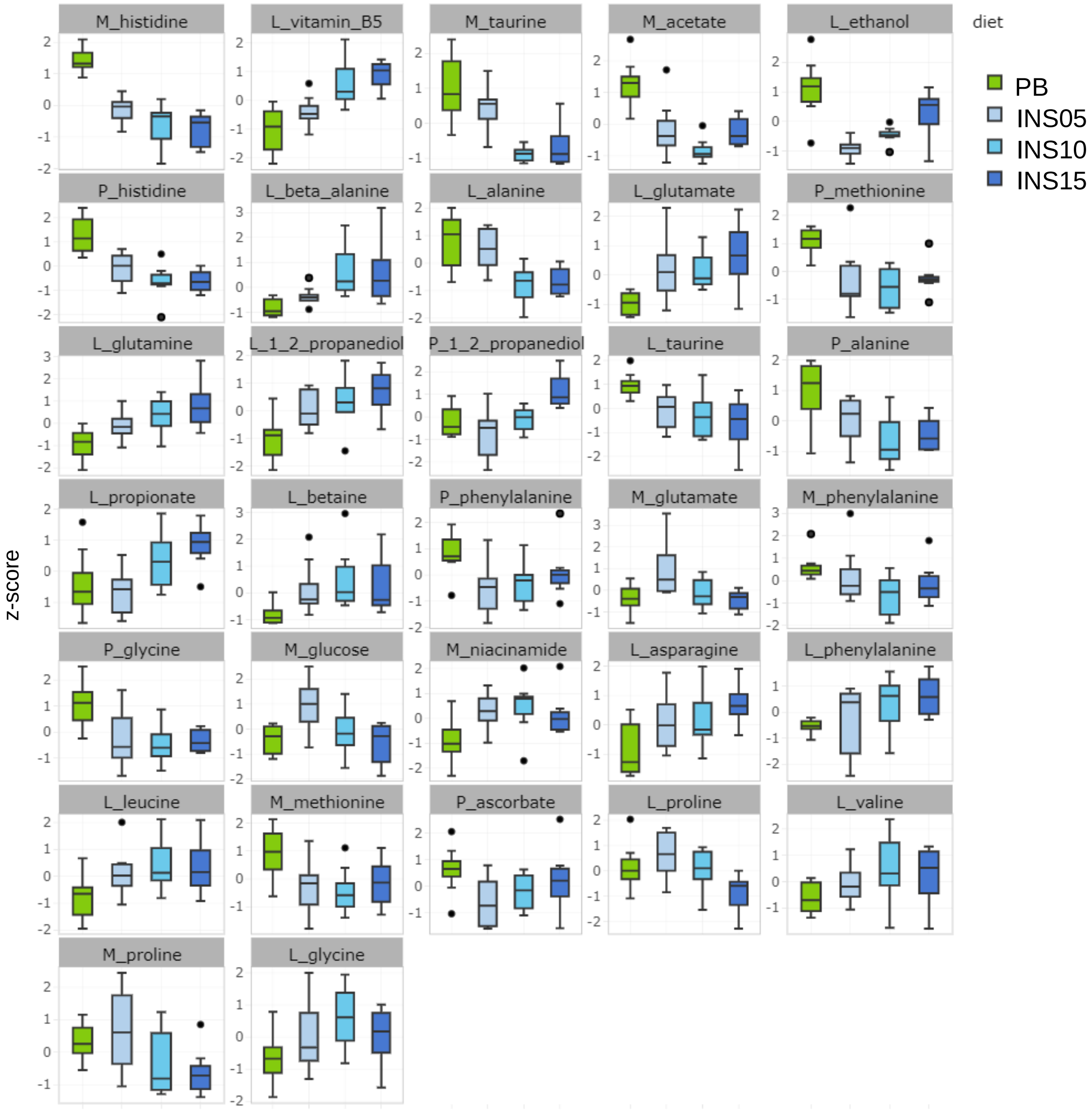

Figure S3. Whisker plots of individual metabolites in plasma (P_), liver (L_) or muscle (M_) for which diet had a statistically significant effect (Kruskal-Wallis analyses, *P*<0.05).

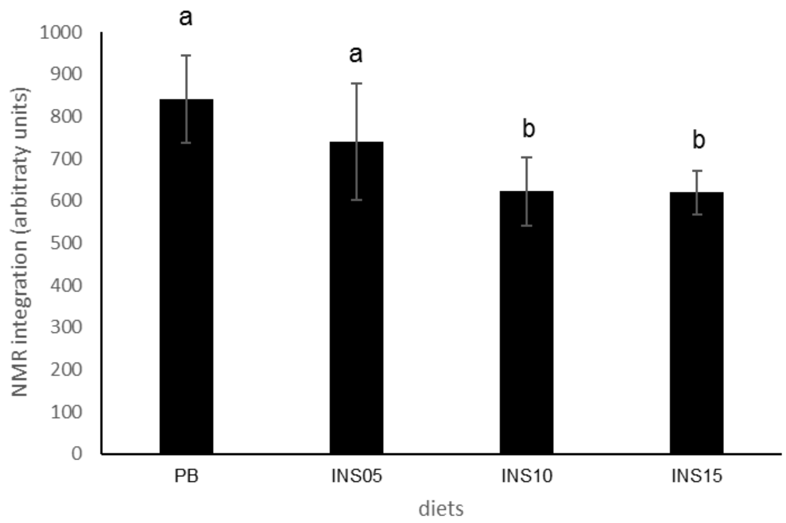

Figure S4. Sum of the intensities of 6 of the free essential amino acids quantified using ^{1}H-NMR spectra of muscle extracts (isoleucine, leucine, valine, methionine, histidine, phenylalanine). Mean of 9 replicates. Vertical bars represent standard deviation. Mean accompanied by the same letter are not significantly different according to Tukey's test (P<0.05)

Figure S5: Representative 500 MHz ^{1}H-NMR spectra of plasma of trout fed INS15 diets. Panels represents zoom in. Letters in brackets describe the pattern multiplicity (s, singlet; d, doublet; t, triplet; q, quadruplet; m, multiplet)

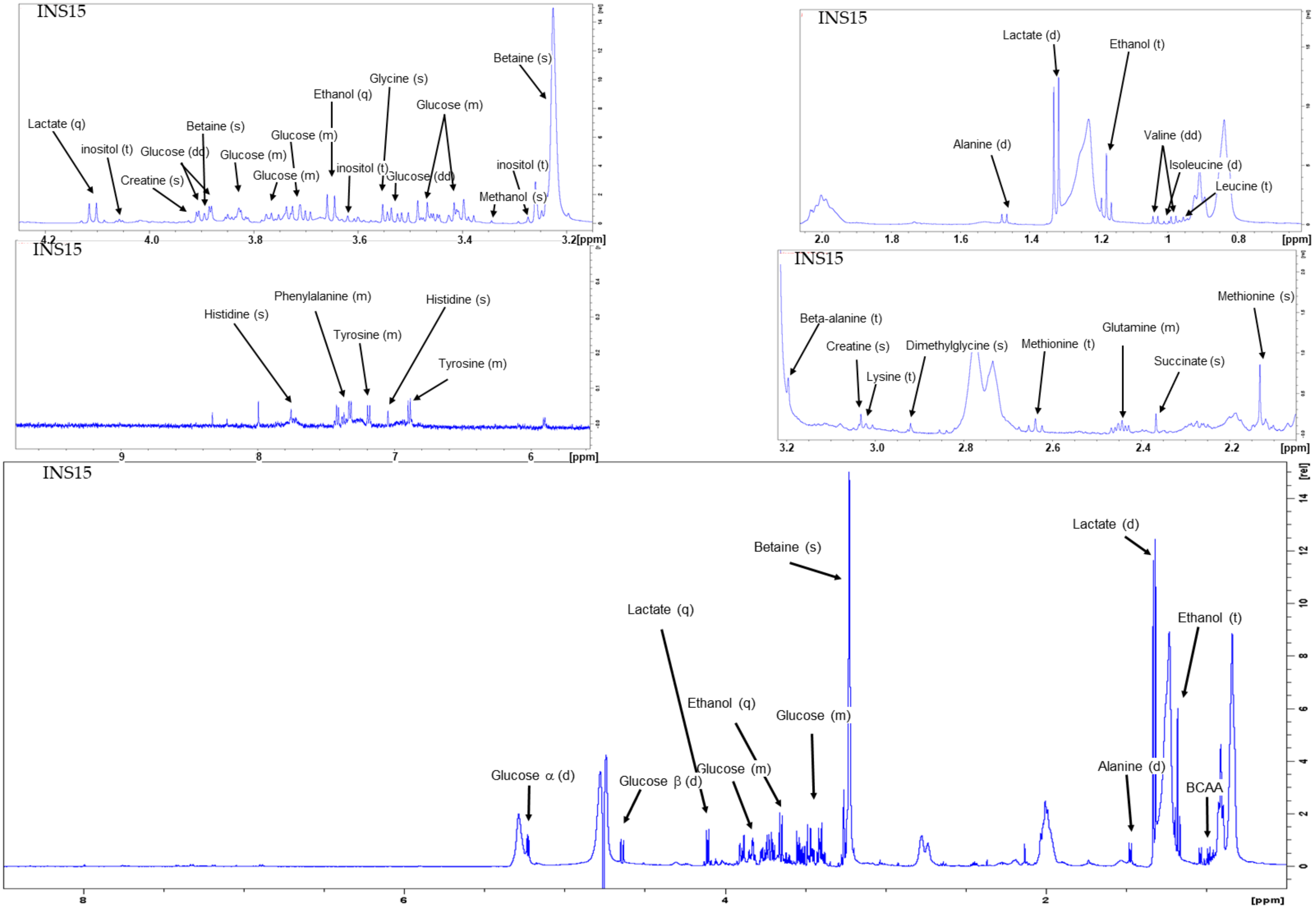

Figure S6: Representative 500 MHz ^{1}H-NMR spectra of liver polar extract of trout fed INS15 diets. Panels represents zoom in. Letters in brackets describe the pattern multiplicity (s, singlet; d, doublet; t, triplet; q, quadruplet; m, multiplet)

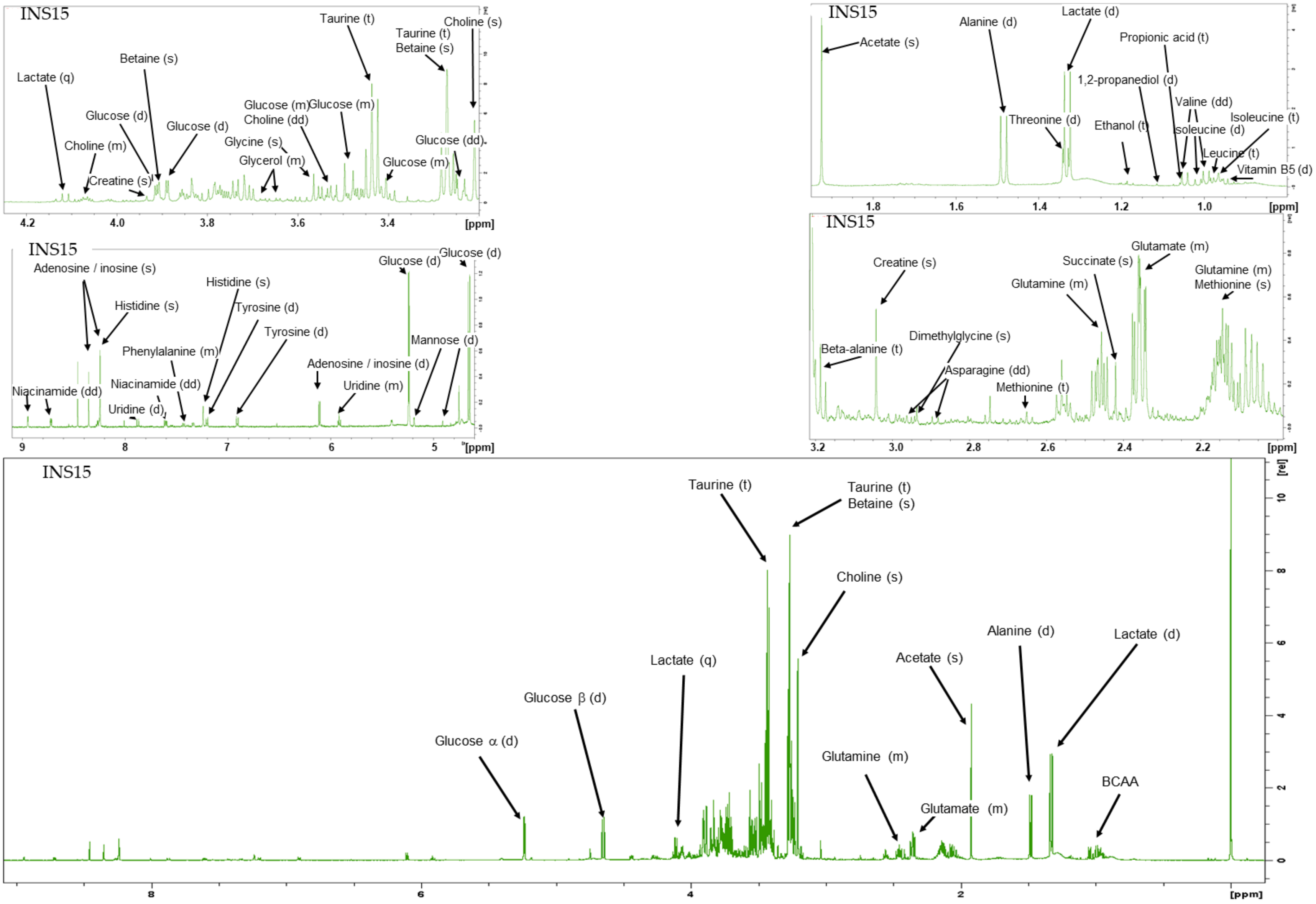

Figure S7: Representative 500 MHz ^{1}H-NMR spectra of muscle polar extract of trout fed INS15 diets. Panels represents zoom in. Letters in brackets describe the pattern multiplicity (s, singlet; d, doublet; t, triplet; q, quadruplet; m, multiplet)

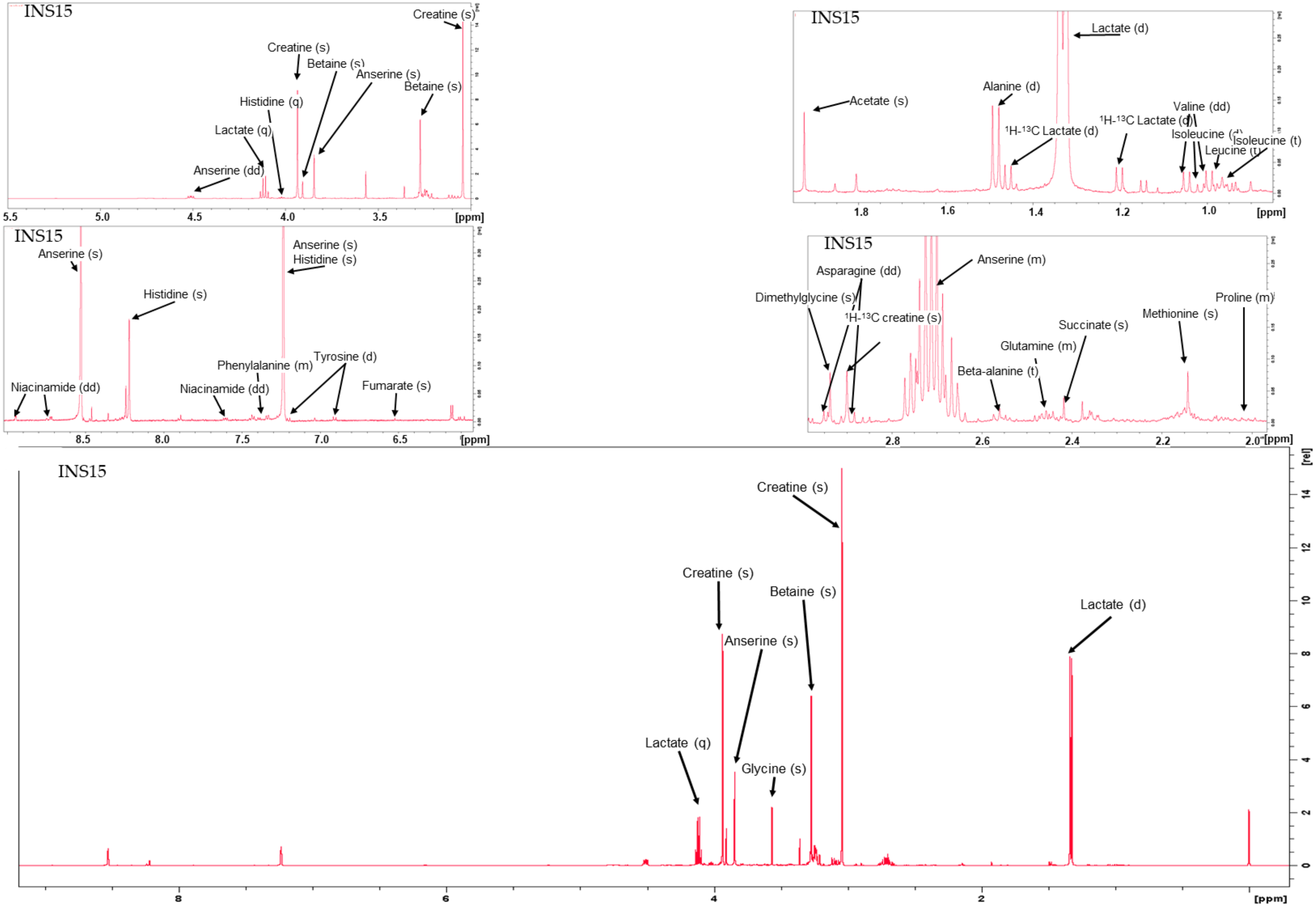

Table S1: Amount of feed consumed during the fish growth period, nitrogen intake and gain for the four diets. Mean ± SD of 9 replicates.

Diet	Feed consumed over the period (g)	N intake ($g.j^{-1}$)*	N gain ($g.j^{-1}$)
PB	5844 ± 289	1.12 ± 0.02 a	0.45 ± 0.02
INS05	5811 ± 139	1.03 ± 0.01 b	0.46 ± 0.01
INS10	6501 ± 212	1.02 ± 0.02 b	0.48 ± 0.01
INS15	6582 ± 192	1.00 ± 0.01 b	0.49 ± 0.01

* statistically significant (ANOVA, P=0.00006)

For the variable significantly affected by diet according to ANOVA, means ± SD accompanied by the same letters are not significantly different (Tukey's test, P<0.05)

Table S2: NMR annotation table of compounds in feed polar extract (F), and of metabolites in trout plasma (P), and liver (L) or muscle (M) polar extracts. Extracts were pH adjusted at apparent pH 6. Underlined F, P, L or M letters indicate the resonance pattern integrated for quantification.

Metabolite/compound	ChEBI ID§	^{1}H Chemical shift in ppm (multiplicity)	Feed, Plasma and Tissues			
Amino acids, dipeptides						
alanine	16449	1.48 (d)	<u>F</u>	<u>P</u>	<u>L</u>	<u>M</u>
		3.78 (q)		P		M
anserine	18323	2.7 (m)				M
		3.09 (dd)				<u>M*</u>
		3.24 (m)				M
		3.84 (s)				M
		4.51 (d)				M
		7.23 (s ou d)				M
		8.53 (s ou d)				M
asparagine	22653	2.87 (dd)			<u>L*</u>	<u>M*</u>
		2.97 (dd)			L	M
		4.02 (dd)				M
aspartic acid	22660	2.68 (dd)			<u>L*</u>	
		2.83 (dd)	<u>F*</u>		L	
		3.91 (dd)				
beta-alanine	29987	2.56 (t)			<u>L*</u>	<u>M</u>
		3.18 (t)		<u>P*</u>	L	
glutamic acid	18237	2.09 (m)			<u>L</u>	
		2.36 (m)			<u>L</u>	<u>M*</u>
		3.76 (dd)				
glutamine	28300	2.13 (m)		P	L	M
		2.45 (m)		<u>P</u>	<u>L</u>	<u>M (2.47)</u>
		3.77 (t)		P		
glycine	15428	3.55 (s)		<u>P</u>	<u>L (3.57)</u>	<u>M (3.57)</u>
histidine	27570	3.23 (dd)				
		3.30 (dd)				M
		4.01 (dd)				<u>M*</u>
		7.05 (d)		<u>P</u>	<u>L (7.23)</u>	M (7.23)
		7.75 (d)		P	L (8.24)	M (8.21)
isoleucine	24898	0.93 (t)	<u>F*</u>	P	L	<u>M (0.95)*</u>
		1.01 (d)	F	<u>P</u>	<u>L*</u>	M
		1.25 (m)				
		1.98 (m)				
		3.67 (d)		P	L	
leucine	25017	0.96 (t)	<u>F*</u>	<u>P*</u>	<u>L *</u>	<u>M*</u>
		1.71 (m)	F	P	L	M

		3.73 (m)				
lysine	25094	1.49 (m)	F			
		1.73 (m)	F	P		
		1.91 (m)	F	P		
		3.03 (t)	F	P*		
		3.76 (t)				
methionine	16811	2.13 (s)	F	P	L	M
		2.64 (t)	F	P	L*	M
		3.85 (m)	F			
proline	26271	2.01 (m)	F*		L	M*
		2.08 (m)	F			M
		2.35 (m)	F			
		3.37 (m)	F			
		4.18 (dd)			L	
pyroglutamic acid	16010	2.03 (m)				
		2.40 (t)	F			
		2.51 (m)	F			
		4.19 (q)	F			
sarcosine	15611	2.75 (s)			L	
		3.62 (s)			L	
serine	17822	3.85 (dd)				
		3.96 (m)		P (3.94)*	L*	
threonine	26986	1.33 (d)	F		L*	
		3.6 (d)	F		L	
		4.27 (q)	F		L	
tyrosine	18186	3.06 (dd)				
		3.21 (dd)				
		3.94 (dd)				
		6.90 (d)	F	P	L	M
		7.19 (d)	F	P	L	M
phenylalanine	28044	3.12 (dd)				
		3.29 (dd)				
		3.99 (dd)				
		7.37 (m)	F*	P*	L*	M*
valine	27266	0.99 (d)	F	P	L	M
		1.04 (d)	F	P	L	M
		2.28 (m)		P	L	M
		3.61 (d)	F	P	L	M
Organic acids						
acetic acid		1.92 (s)			L	M
citric acid	30769	2.57 (dd)	F			
		2.69 (dd)	F			
fumaric acid	18012	6.52 (s)	F		L	M

lactic acid	24996	1.33 (d)	F	P	L*	M
		4.11 (q)	F*	P	L	M
malic acid	6650	4.30 (dd)	F			
		2.68 (dd)	F			
		2.37 (dd)	F			
propionic acid		1.06(t)			L*	
		2.18 (q)				
succinic acid		2.37 (s)	F(2.42)	P	L(2.42)	M(2.42)
Sugars						
glucose	17234	3.24 (t)		P	L	
		3.55-3.37		P	L	M
		3.86-3.69		P	L	M
		3.89 (d)		P	L	M
		3.91 (d)		P	L	
		4.64 (d)	F	P	L	M
		5.23 (d)	F	P	L	M
mannose	37684	3.39 (m)				
		3.57 (t)				
		3.66 (m)				
		3.96-3.71				
		4.91 (d)			L	
		5.18 (d)			L	
stachyose	17164	3.53 (t)				
		3.58 (dd)				
		4.17-3.65 (m)	F			
		4.23 (d)	F			
		5.00 (m)	F			
		5.44 (d)	F			
sucrose	17992	3.48 (t)	F			
		3.57 (dd)				
		3.68 (s)	F			
		3.77 (t)				
		3.83 (m)	F			
		3.88 (m)				
		4.06 (t)	F			
		4.22 (d)	F			
		5.42 (d)	F			
Alcohols, cyclitols						
1,2-propanediol	16997	1.14 (d)	F	P	L*	
ethanol	16236	1.18 (t)		P	L*	
		3.66 (q)		P	L	
glycerol	17754	3.56 (m)	F		L	
		3.66 (m)	F*		L*	

		3.79 (m)	F			
inositol	17268	3.28 (t)		P		
		3.53 (dd)		P		
		3.63 (t)		P		
		4.05(t)		P		
Amines and N-containing compounds						
betaine	17750	3.26 (s)	F	P	L	M
		3.90 (s)	F	P	L	M
choline	15354	3.21 (s)	F		L	
		3.53 (t)	F		L	
		4.07 (m)			L	
creatine	16919	3.04 (s)		P	L	M
		3.93 (s)		P	L	M
dimethylglycine	17724	2.94 (s)		P (2.92)	L	
ethanolamine	16000	3.14 (t)	F			
		3.81 (t)				
phosphocholine	18132	3.23 (s)	F			
taurine	15891	3.27 (t)			L	
		3.44 (t)			L	M
Nucleobases, nucleosides						
adenosine / inosine	16335/ 17596	3.85 (dd)				
		3.93 (dd)				
		4.28 (q)			L	
		4.44 (q)			L	
		6.10 (d)			L	
		8.24 (s)			L	
		8.35 (s)			L	
uracil	17568	5.80 (d)	F			
		7.54 (d)	F			
uridine	16704	3.81 (dd)				
		3.92 (dd)				
		4.14 (m)				
		4.24 (t)			L	
		4.36 (t)			L	
		5.92 (dd)	F		L	
		7.89 (d)	F		L	
Vitamins						
ascorbate	22651	3.77 (m)				
		4.00 (t)				
		4.50 (d)		P		
niacinamide	17154	7.59 (dd)			L	M
		8.23 (dd)				

		8.70 (dd)	L	M
		8.92 (s)	L	M
vitamin B5	7916	0.90 (s)	L	M
(pantothenic acid)		0.93 (s)	L*	M*
		2.40 (t)		
		3.38 (d)		
		3.43 (t)		
		3.50 (d)		
		3.97 (s)		

§ All metabolites/compounds with carboxylic group are not indicated with the ChEBI ID of the carboxylate form, except for ascorbate. All metabolites/compounds with carboxylic group and amino group are not indicated with the ChEBI ID of the zwitterion form.

* indicates that only a part of the multiplet is integrated

Numbers in brackets in the Feed and Tissues columns indicate that resonance exhibited a slight shift compared to other sample types.

Table S3: MSI annotation levels for metabolites/compounds in the NMR spectra of feed polar extract, trout plasma, liver and muscle polar extracts, according to Sumner et al. (2007).

	Feed	Plasma	Liver	Muscle
1,2-propanediol	1	2	2	
acetate			1	2
adenosine / inosine			3	
alanine	1	2	1	1
anserine				1
ascorbate		2		
asparagine			1	1
aspartate	1		2	
beta-alanine		2	1	1
betaine	1	2	1	1
choline	1		1	
citrate	1			
creatine			2	1
dimethylglycine		2	2	
ethanol	1		2	
ethanolamine				
fumarate	2		2	2
glucose	1	2	1	1
glutamate			1	1
glutamine		2	1	1
glycerol	1		2	
glycine		2	1	1
histidine		2	1	1
inositol		2		
isoleucine	1	2	1	1
lactate	1	2	1	1
leucine	1	2	1	1
lysine	1	2		1
malate	1			
mannose			2	
methionine	1	2	2	1
niacinamide			1	1
phenylalanine	1		1	1
phosphocholine				
proline	1		3	1
propionate			2	
pyroglutamate	1			
sarcosine			2	
serine		2	1	
stachyose	1			
succinate	1	2	2	2
sucrose	1			
taurine			1	1
threonine	1		1	
tyrosine	1	2	1	1
uracil	1			
uridine	1		1	
valine	1	2	1	1

vitamin B5	2	2

Sumner et al. *Metabolomics*, *3*(3), 211-221.

Table S4: Total amino acid (free amino acids and protein amino acids) concentrations in the four feeds ($g.100g^{-1}$)

	PB	INS05	INS10	INS15
alanine	1.57	1.59	1.62	1.54
arginine	3.08	2.78	2.78	2.63
aspartic acid	3.96	3.71	3.75	3.64
cystine	0.5	0.52	0.49	0.44
glutamic acid	7.94	7.89	7.17	6.53
glycine	1.53	1.56	1.51	1.45
histidine	0.98	0.94	0.94	0.91
isoleucine	1.81	1.72	1.7	1.66
leucine	3	2.87	2.82	2.69
lysine	2.71	2.53	2.57	2.45
methionine	1.12	1.07	1.07	1.09
phenylalanine	1.99	1.87	1.87	1.75
proline	2.01	2.23	2.1	1.37
serine	2.01	1.9	1.78	1.64
threonine	1.7	1.64	1.62	1.48
tyrosine	1.34	1.3	1.34	1.27
valine	1.89	1.86	1.86	1.75
